# Social actors’ perceptions of wildlife: Insights for the conservation of species in Mediterranean protected areas

**DOI:** 10.1007/s13280-021-01546-6

**Published:** 2021-07-12

**Authors:** Ainara Cortés-Avizanda, Henrique M. Pereira, Ellen McKee, Olga Ceballos, Berta Martín-López

**Affiliations:** 1 Departamento de Biología Vegetal y Ecología, Faculty of Biology, University of Seville, Av. Reina Mercedes s/n, 41012 Seville, Spain; 2 Department of Conservation Biology, EBD (CSIC), C/. Americo Vespucio 26, 41092 Sevilla, Spain; 3 Infraestruturas de Portugal Biodiversity-Chair CIBIO-InBIO Centro de Investigacão em Biodiversidade e Recursos Geneticos da Universidade do Porto, Campus Agrário de Vairão, Rua Padre Armando Quintas, nº 7, 4485-661 Vairão, Portugal; 4 German Centre for Integrative Biodiversity Research (iDiv) Halle-Jena-Leipzig, Deutscher Platz 5e, 04103 Leipzig, Germany; 5 Institute of Biology, Martin Luther University Halle-Wittenberg, Am Kirchtor 1, 06108 Halle, Saale Germany; 6 UGARRA, Avda. Carlos III 1, 31002 Pamplona, Spain; 7 Faculty of Sustainability, Leuphana University of Lüneburg, Universtitätsalle 1, 21355 Lüneburg, Germany

**Keywords:** Environmental knowledge, Game species, Predators, Protected area, Scavengers, Steppe birds

## Abstract

**Abstract:**

In the current Anthropocene Era, with numerous escalating challenges for biodiversity conservation, the inclusion of the social dimension into management decisions regarding wildlife and protected areas is critical to their success. By conducting 354 questionnaires in a Mediterranean protected area (the Biosphere Reserve of Bardenas Reales, Northern Spain), we aim to determine sociodemographic factors influencing knowledge levels and perceptions of species and functional groups as, emblematic and threatened. We found that hunters and animal husbandry workers knew more species than other social actors. Additionally, the perception of functional groups as threatened or emblematic differed between social actor groups, with statistically significant associations between perceptions and the characteristics of respondents. Interestingly, we found that although elusive steppe species are globally considered as endangered, these species were the least known by all social actor groups and rarely perceived as emblematic. This research is a novel approach and provides a better understanding of how perceptions can facilitate conservation decisions, particularly regarding endangered species

**Graphic abstract:**

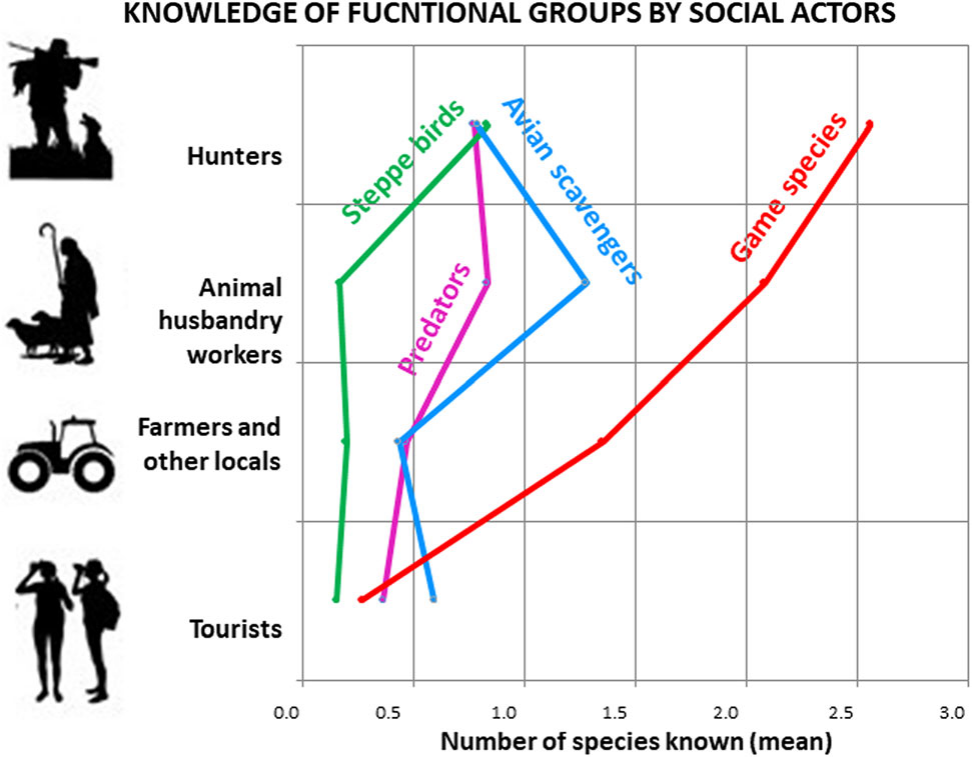

**Supplementary Information:**

The online version contains supplementary material available at 10.1007/s13280-021-01546-6.

## Introduction

In 1968, at the general assembly of the World Conservation Union, Baba Dioum stated ‘…in the end, we will conserve what we love. We will love only what we understand. We will understand only what we are taught’ (Main 2004). This statement highlights the critical need to recognize the importance that social perceptions and knowledge have on conservation outcomes (e.g., Ban et al. 2013; Martín-López and Montes 2015; Bennett et al. 2016, 2017). This is especially true within the current Anthropocene era as numerous challenges for biodiversity conservation linked to anthropogenic drivers continue to escalate (Barnosky et al. 2011; Pereira et al 2012; Lewis and Maslin 2015). Indeed, it is now accepted that conservation is as much about people as it is about species (Mascia et al. 2003). Scientists and policy makers are increasingly recognizing that to successfully preserve biodiversity and protect habitats, it is of critical importance to take into consideration the social perceptions towards wildlife to foster social support (Fischer et al. 2012; Soulé 2013; Marvier 2014; Tallis and Lubchenco 2014; Bennett et al. 2016, 2017; Oldekop et al. 2016; IPBES 2019).

According to Bennett et al. (2016), the evaluation of the sociodemographic characteristics of social actors that determine and underpin perceptions about wildlife and the conservation of particular ecological systems, may ultimately contribute to ensuring the success of conservation with social support. Despite considerable efforts to build an evidence base regarding the integration of human perceptions in biodiversity conservation, research, thus, far has mainly been species specific (e.g., Kellert 1985; Oli et al. 1994; Conforti and Azaavedo 2003; Bhattarai and Fischer 2014). This is the case for certain studies that focus exclusively on specific species of carnivores, ungulates, scavengers, and steppe birds (e.g., Villanúa et al. 2007; Delibes-Mateos et al. 2015; Cortés-Avizanda et al. 2018) and/or focus on a specific social actor (e.g., Pascual-Rico et al. 2020; Martinez-Sastre et al. 2020).

The study of social perceptions of wildlife from a broader perspective (studying multiple social actor groups and species) is necessary in order to avoid polarized discourses that may perpetuate the narratives on human-wildlife conflicts (Lozano et al. 2019). A broader research scope can also avoid the tendency to focus exclusively on emblematic species (i.e., species with great symbolic value in a place, which are recognizable for the people who visit the place) such as large mammals, which generally receive considerable research and social attention in contrast to rare or more elusive species (Urbanik 2012). Increasing the research scope beyond a single species or a specific social actor group can contribute to the design of holistic conservation programs that consider the complexity of ecological and social systems.

Protected areas within Mediterranean biomes contain many species experiencing population declines due to a variety of anthropogenic drivers, such as land-use change, direct and indirect persecution, and climate change (Groom et al. 2006; Díaz et al. 2019; Leberger et al. 2020). Here, the accommodation of different perceptions, interests, and needs of various social actors into wildlife conservation programs may result in improved conservation success (Soulé 2013; Marvier 2014). In this context, our primary goal was to identify and characterize the perceptions held by different social actor groups of a range of wildlife species found within the Biosphere Reserve of Bardenas Reales (northern Spain). We aimed to provide an understanding of the factors influencing the knowledge and perceptions of species held by the different social actors: hunters, tourists, animal husbandry workers, and local inhabitants (including traditional crop farmers) that live in the neighborhoods bordering the protected area. Following this, we provide guidelines for future policy decisions regarding biodiversity conservation in Mediterranean biomes. We specifically aim to determine (i) which individual species and functional groups of species are the least known by each social actor group, (ii) which species are perceived as emblematic and threatened, and (iii) which sociodemographic factors influence social perceptions of species. By understanding which sociodemographic characteristics determine social actors’ perceptions of species, we ultimately intend to provide insights for conserving biodiversity in this region and other protected areas in the Mediterranean Basin. This wider analysis elucidating the perceptions of various social actor groups towards main functional groups is key in order to foster novel conservation strategies to protect and recover vulnerable Mediterranean species with public support.

## Materials and methods

### Study area

**Fig. 1:**
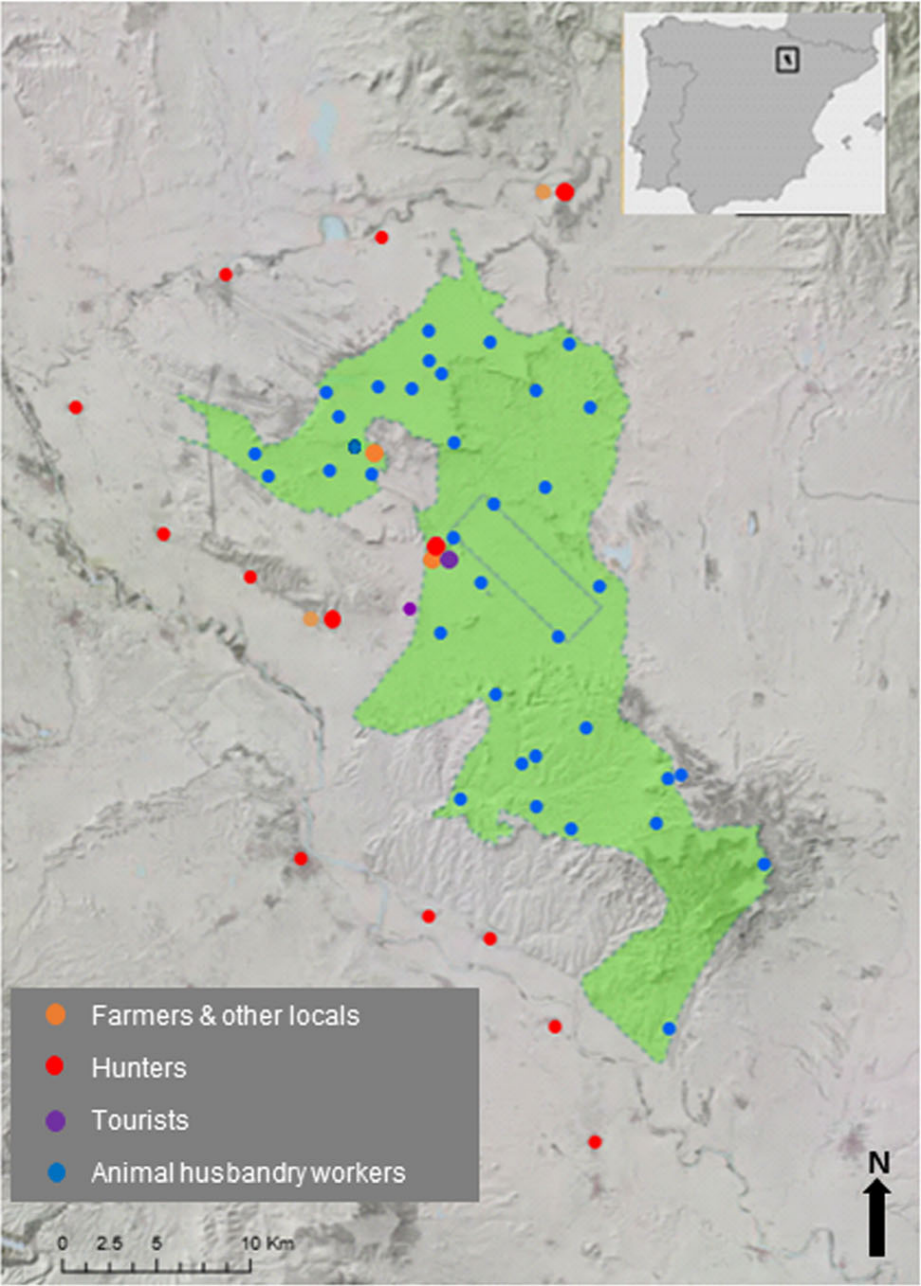
Study area of the Bardenas Reales Natural Park and Biosphere Reserve (northern Spain) with the locations of where the questionnaires were conducted with different social actor groups. Questionnaires with animal husbandry workers were performed widely within the protected area whereas the rest of the social actor groups were questioned at particular sites (e.g., scenic viewpoints, information center of the park and nearest towns, see details in Supplementary material) because no human permanent settlements exist inside the Protected Area. The Protected Area is represented in a green color with a dashed line for the borders. The rectangle delimits a military area. *Source* CLC2000-100 m version 17 (12-2013)

Bardenas Reales Natural Park and Biosphere Reserve encompasses around 50 000 ha in northern Spain (Fig. 1). This protected area has no human settlements as only traditional agriculture and livestock keeping are permitted (see details in Cortés-Avizanda et al. 2009, 2018). This contrasts with surrounding areas that hold dense human settlements and intensive farming areas (Cortés-Avizanda et al. 2009, 2015; Arrondo et al. 2020). Interestingly, this area constitutes a singular region because it is the breeding habitat of several species representative of the Mediterranean Basin. For instance, steppe birds such as the cryptic and critically endangered Dupont’s lark (*Chersophilus duponti*) and the pin-tailed sandgrouse (*Pterocles alchata*), large birds of prey such as the golden eagle (*Aquila chrysaetos*) and the marsh harrier (*Circus aeruginosus*) and avian scavengers such as the endangered Egyptian vulture (*Neophron percnopterus*) (Cortés-Avizanda et al. 2009, 2018). Furthermore, the region also contains carnivores such as foxes (*Vulpes vulpes*), game species such as the red-legged partridge (*Alectoris rufa*), the wild boar (*Sus scrofa*), and keystone species such as the wild rabbit (*Oryctolagus cuniculus*) (Cortés-Avizanda et al. 2015).

### Data collection

We conducted the data sampling in two phases: semi-structured interviews and questionnaires (Young et al. 2018). We first conducted semi-structured interviews (*n* = 10) with traditional farmers, hunters, animal husbandry workers, and wildlife managers in the study area to identify: (i) biodiversity and social actors present in the study area (Online Appendix A) and (ii) motivations for the conservation of the biodiversity. We applied a snowball sampling technique to identify additional respondents, i.e., we asked respondents to name others who could be contacted for their knowledge about this protected area and its biodiversity. All the interviews were conducted with the signed consent of interviewees and digitally recorded for later transcription and codification. To adhere to the ethical principles of conducting interviews, we followed three ethical principles: (i) all respondents were fully informed about the scope and main goal of the research, as well as further use of the information collated and dissemination of results; (ii) before undertaking the interviews, we asked for the respondents’ informed voluntary consent in a written form, and (iii) we ensured anonymity and privacy of the interviewees. The main goal of the semi-structured interviews was to design the structure and content of the questionnaire. In the second step, we conducted 354 face-to-face questionnaires in the protected area during spring 2015. The respondents were randomly selected while they were visiting the park. We sampled individuals over 18 years old belonging to the four main social actor groups identified in the previous interviews; animal husbandry workers (10.2% of respondents), hunters (18.6%), local inhabitants (34.5%), and tourists (36.7%) (Online Appendix A). For each social actor group, we estimated a representative sample size of respondents at a 95% confidence level, with a sampling error ranging between 4.4 and 6.0% (Table S1 in  Online Appendix A).

Based on the information gathered through the semi-structured interviews, we designed a structured questionnaire that was organized in five sections. In the first section, we collected information on the sociodemographic characteristics of respondents (e.g., residence, age, and gender). In the second section, we collected information about the respondents’ environmental behavior (e.g., frequency of visits to the area per year, number of other protected areas visited in the last year). The third section targeted respondents’ environmental knowledge regarding the protected area and the conservation status of its biodiversity. In the fourth section, we gathered information on respondents’ knowledge about the species living in the protected area. The final section focused on respondents' perceptions regarding the threat status and emblematic nature of the main functional groups of species: steppe birds, avian scavengers, game species (considering both birds and mammals), and predators (considering both raptors and terrestrial carnivores) (i.e., based on interviewed' perceptions and knowledge, Online Appendix B). We used open-ended questions for obtaining information on the respondents’ knowledge of species diversity and the threat status of species. The answers of the open-ended questions were assigned to four functional groups of species: game species, scavengers, steppe birds, and predators. Then, in the last section, we used semi-opened-ended questions to determine whether the functional groups of species were considered emblematic and threatened. Questions from the fourth and fifth sections comprised the dependent variables, whereas questions from the first three sections comprised the set of explanatory variables (Table 1). Online Appendix B presents the complete questionnaire. To conduct the questionnaire, we also adhered to the standard ethical principles of social research: the principle of full disclosure, the principle of prior informed voluntary consent (which was verbally obtained), and the principle of confidentiality.

**Table 1:** Explanatory variables used in the redundancy analysis. *PA* protected area

Variables	Type	Attributes
Social actor		
Animal husbandry workers	Dummy	1 = Livestock keeper, 0 = otherwise
Farmers and other locals	Dummy	1 = Local actor than livestock keeper, 0 = otherwise
Hunters	Dummy	1 = Hunter, 0 = otherwise
Tourists	Dummy	1 = Tourist, 0 = otherwise
Sociodemographic		
Gender	Dummy	1 = Male, 0 = Female
Age	Continuous	Ln (age in years)
Residence time	Continuous	Ln (years of residence in Bardenas)
Environmental behavior		
Frequency of visits to Bardenas PA	Continuous	Number of visits per year
Number of PAs visited in the last year	Continuous	Number of visits in the last year, i.e., 2014
Environmental knowledge		
Knowledge of Bardenas as a PA	Dummy	1 = respondent knows that Bardenas is a PA, 0 = otherwise
N species known		Number of species present in Bardenas PA known by respondents

### Data analysis

To examine the degree of knowledge between different social actors regarding the species present in the Bardenas Reales protected area (i.e., number of species known), we fit a Generalized Linear Model (GLM) considering the number of species known (Poisson distribution; log link function) as the response variable with two explanatory variables: (i) Social actor (four levels: animal husbandry workers, hunters, farmers-local inhabitants, and tourists) and (ii) functional group of species (game species, scavengers, steppe birds, and predators). We also fitted the interaction Social actor*species in order to evaluate potential dissimilarities in social actor knowledge and species known. We used the MuMIn package (Barton 2019) for the GLM analysis. Model selection was made following the Akaike Information Criterion corrected for small sizes (AICc, Sugiura 1978). All analyses were performed in RStudio-3.6.3 (RStudioTeam 2018).

In addition, to detect the sociodemographic factors underpinning social actor perceptions of the threat status and emblematic nature of species, we conducted two redundancy analyses (RDA): (1) emblematic and (2) threatened species. A Monte Carlo permutation test (500 permutations) was conducted to test the significance of the explanatory variables in influencing the mentioned perceptions of the four defined groups. We included the social actor group (i.e., animal husbandry workers, hunters, local inhabitants, and tourists, see Table S1 in Online Appendix A), sociodemographic characteristics, environmental behavior, and environmental knowledge as explanatory variables (Table 1). The standardized coefficients of the explanatory variables were used to indicate the relative importance of these variables in explaining the dependent variables. Continuous variables were log-transformed prior to the RDA in order to minimize heteroscedasticity.

## Results

**Fig. 2:**
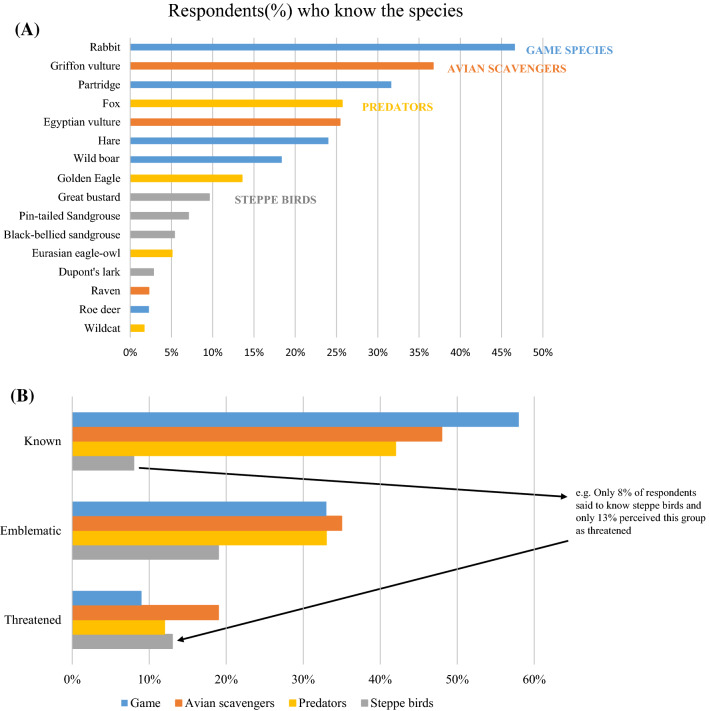
Percentage of respondents who have a knowledge of each game species, avian scavengers, steppe birds, and predators: **a** knowledge of individual species and **b** knowledge of the four groups of species. Note that figure A shows only the species that have been cited more than 10 times and the percentage (%) was calculated on the total of responses

Social actors mentioned the wild rabbit the most (47% of respondents), followed by the griffon vulture (37%), the red-legged partridge (32%), the fox (26%), and the Egyptian vulture (25%) (Fig. 2a). In general, while steppe birds are the least mentioned (only 8% of respondents), game species and avian scavengers were mentioned by 58% and 48% of respondents, respectively (Fig. 2b). In addition, while game species, avian scavengers, and predators were considered as emblematic species by more than 33% of respondents, less than 20% of respondents considered these groups as threatened (Fig. 2b). Interestingly, steppe birds were mentioned by only 8% of the respondents with 19% of respondents considering steppe birds as charismatic and 13% considering them as threatened (Fig. 2b).

**Fig. 3:**
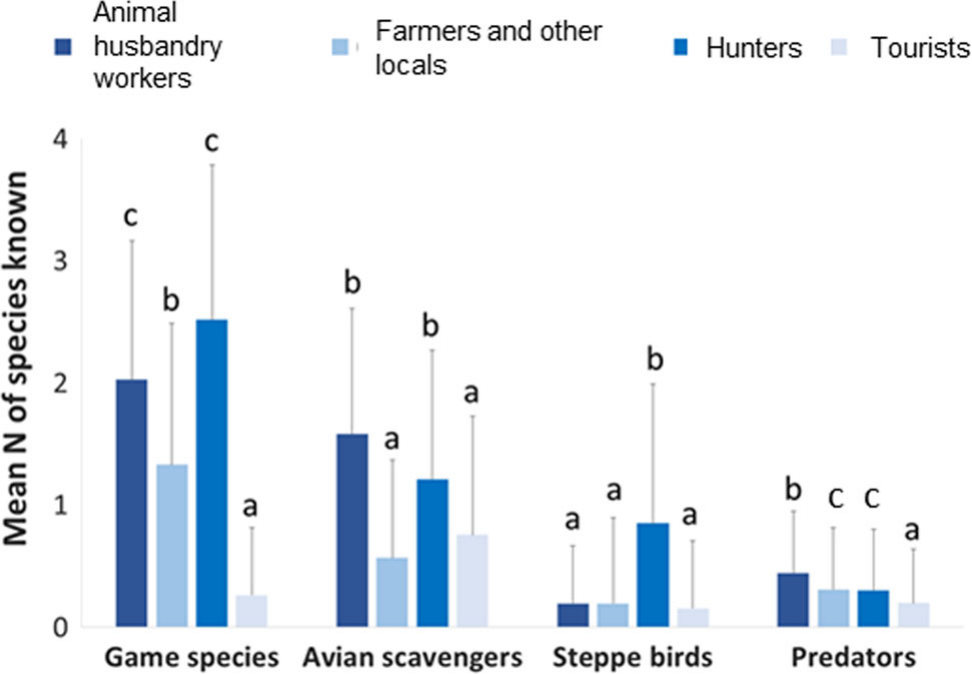
Knowledge of species inhabiting the Bardenas Reales Natural Park held by different social actors. Bars and whiskers indicate the mean value of the number of species mentioned and the standard deviation, respectively. Different letters (a, b or c) indicate significant differences from one social actor group to another according to Dunn's multiple comparison test (*p* value < 0.01)

The knowledge regarding the species of each functional group differed significantly between social actor groups (see Table 2, Fig. S1 in Online Appendix A). Overall, hunters and animal husbandry workers were more knowledgeable of species than tourists particularly in the case of game species and avian scavengers (Fig. S1 in Online Appendix A). In addition, the interaction between social actors and species determined that that hunters named significantly more game species and steppe birds than other social actor groups (See Table 2, Figs. 2 and 3), while animal husbandry workers and farmers identified the greatest number of avian scavengers and predators (see Table 2, Fig. S1 in Online Appendix A). Tourists knew the fewest number of species within each functional group apart from avian scavengers and predators. Interestingly, steppe birds were the least known species functional group by all social actor groups except hunters (see Table 2, Fig. 2).

**Table 2:** Estimates and confidence intervals from the best GLM for the response variable number of species known in Bardenas Reales Natural Park and Biosphere Reserve

	Estimate	Std. error	CI 2.5%	CI 97.5%
(Intercept)	0.940	0.077	0.786	1.087
Predators	− 1.198	0.160	− 0.483	0.061
Avian scavengers	− 1.179	0.159	− 0.853	− 0.424
Steppe birds	− 1.123	0.155	− 2.632	− 1.902
Social actor animal husbandry workers	− 0.206	0.139	− 1.521	− 0.893
Social actor farmers & other locals	− 0.638	0.109	− 1.499	− 0.876
Tourists	− 2.252	0.186	− 1.435	− 0.826
Predator: animal husbandry workers	0.282	0.269	− 0.252	0.805
scavengers: animal husbandry workers	0.690	0.245	− 0.299	0.571
Steppe: animal husbandry workers	− 1.403	0.452	0.960	2.038
Predator: farmers & other locals	0.135	0.222	0.208	1.172
Scavengers: farmers & other locals	0.043	0.224	− 0.396	0.483
Steppe: farmers & other locals	− 0.797	0.268	1.470	2.484
Predator: tourists	1.493	0.275	− 2.391	− 0.590
Scavengers: tourists	1.967	0.258	− 1.337	− 0.282
Steppe: tourists	0.563	0.320	− 0.077	1.185

*S.E.* standard error, *CI* confidence interval

The RDA showed that a statistically significant association exists between the perception of the four functional groups as emblematic and threatened and the characteristics of respondents, i.e., social actor group, environmental behavior, knowledge, and sociodemographic characteristics (*p* value < 0.0001 in both RDAs, from 500 permutations). In both RDAs, the two main axes explained more than 80% of the total variance (Table 3). In the first RDA conducted with the scores on the perception of species as emblematic, we found that the first axis (58.5% of variance) explained the perceptions of game species (in the positive scores) and avian scavengers (in the negative scores) as emblematic species. The perception of game species as emblematic species was associated with male hunters who frequently visit the study area and have lived in the region for a long time (46.3 (± 13.1) years). The perception of avian scavengers as emblematic species was associated with local inhabitants and tourists, mostly women, who have visited other protected areas previously. The second axis (23.1% of variance) showed an association between the perception of avian scavengers and predators as emblematic species with tourists who frequently visited the protected area (Table 3).

**Table 3:** Results of the redundancy analysis (RDA). Bold values represent those groups of species (dependent variables) with higher squared cosines for axes 1 and 2 in both RDAs and those explanatory variables with a standardized coefficient > 0.1

	Emblematic species		Threatened species	
	Axis 1	Axis 2	Axis 1	Axis 2
Dependent variables				
Game species	**1.481**	0.355	**0.633**	− **0.469**
Avian scavengers	− **0.692**	− 0.012	0.516	**0.501**
Steppe birds	− 0.020	− 0.167	**0.955**	0.040
Predators	− 0.533	**1.007**	0.030	0.005
Explanatory variables				
* Social actor group*				
Animal husbandry workers	0.021	0.082	− 0.009	− 0.032
Farmers and other Locals	− **0.208**	− **0.302**	− 0.147	0.000
Hunters	**0.520**	0.075	**0.317**	− 0.071
Tourists	− **0.314**	**0.228**	− **0.148**	**0.102**
* Environmental behavior*				
N protected areas visited	− 0.139	0.055	**0.128**	0.060
Frequency of visits	0.258	**0.136**	0.229	− 0.088
* Environmental knowledge*				
Bardenas as a protected area	0.014	0.109	0.039	0.014
N species known	0.282	− 0.007	**0.347**	**0.073**
* Sociodemographic*				
Male	**0.270**	− 0.016	0.134	− 0.069
Age	0.111	0.154	0.035	**0.112**
Residence time	**0.314**	− 0.124	**0.079**	− 0.057
RDA statistics				
Eigenvalue	0.355	0.140	0.190	0.057
Variance explained (%)	58.517	23.118	66.323	19.805
Cumulative variance (%)	58.517	81.635	66.323	86.129

On the other hand, the first axis (66.3% of variance) of the RDA conducted with the scores on the perception of species as threatened showed that hunters perceived game species and steppe birds as threatened. In addition, perceptions of game species and steppe birds as threatened species were also associated with those older respondents who had worked in the area for a longer time, had higher rates of visits to other protected areas, and held a greater knowledge of the species within the area (Table 3). Along the second axis (19.8% of variance), the perception of avian scavengers as threatened species was associated with elderly tourists who held a greater knowledge of the species within the area (Table 3).

## Discussion

Including the human dimension in conservation management may improve the likelihood of success (Bennett 2016). By exploring the understandings that may exist in society regarding differences in the perceptions of species as emblematic and threatened, novel conservation actions that have social support can be fostered (e.g., Morales-Reyes et al. 2018; Duriez et al. 2019; Lambertucci et al. 2021). Defining social perceptions towards species can help integrate the human dimension by identifying which species should be targeted in conservation programs for example in education agendas. Our findings reveal that knowledge levels and perceptions of species differed between social actor groups and thus, form, an evidence base to help foster increased social support for conservation through targeted actions.

### Knowledge of species

Our results reveal that knowledge of different species varies significantly between social actor groups. This suggests that people within each social actor group may have developed different knowledge based on their experiences in nature. For example, we have found that hunters know more game species than the other social actor groups. In addition, we found that animal husbandry workers know predators to a greater degree than other social actor groups, which suggests that knowledge of species could be derived from detrimental experiences with biodiversity since animal husbandry workers experience conflicts with predators due to livestock loss (e.g., Morales-Reyes et al. 2019). Additionally, we found that knowledge can be built from positive interactions as hunters and animal husbandry workers hold a greater knowledge of avian scavengers compared with other social actor groups. This can be explained as these social actor groups are likely to hold higher levels of local functional and experiential knowledge on how scavengers provide the service of carcass removal from their experiences in the field (Cortés-Avizanda et al. 2015; Morales-Reyes et al. 2019). Interestingly, we have found that steppe birds were the least known functional group to all social actor groups except hunters who in general know more species.

Interestingly, we found that there is a positive relationship between knowledge of species and age. This was the case for animal husbandry workers who had an average age of 53 years in comparison to local inhabitants who named fewer species and who had an average age of 41.5 years old, suggesting that age is a key factor in determining knowledge of species (see also Table 3). These results are in line with previous research focused on other localities in Spain that found that elderly hunters and animal husbandry workers hold more ecological knowledge than their younger counterparts (Oteros-Rozas et al. 2013; Cortés-Avizanda et al. 2018; Morales-Reyes et al. 2018). It should be noted that the correlation between age and experience on knowledge levels remains limited to certain systems; therefore, more research is required in order to get comprehensive applicability.

These findings suggest that blanket education programs may be ineffective if they do not target the knowledge gaps, needs, and interests of each social actor and age group. For example, in our study area, environmental education programs should be focused on young people to build knowledge levels in the younger age groups. Training evidenced by research on knowledge gaps should be given to educators (e.g., trained and experienced tour guides) to tailor programs to more effectively fill knowledge gaps. Building the knowledge base of the social actor and age groups with lower knowledge levels will likely improve conservation support as previous research shows how experience‐based and local ecological knowledge can promote positive perceptions of functional groups; for example, Morales-Reyes et al (2018) demonstrate this to be the case for farmers and their perceptions on scavengers.

### Perceptions of species as emblematic and threatened

Our results found that respondents perceived more species as emblematic as threatened (Fig. 2). Previous research has shown that people are more willing to support conservation actions directed at species that they perceive as emblematic rather than endangered (Colléony et al. 2017; Morales-Reyes et al. 2018; Duriez et al. 2019; García-Alfonso et al. 2019). We found that steppe birds were the least known species and were rarely perceived as emblematic by all social actor groups which may flag a potential lack of social support for the conservation of this group of species. However, we found that steppe birds were the group perceived as the most threatened after avian scavengers (Fig. 3). This finding highlights the need for educational programs focused on the need for more conservation actions directed at endangered and threatened species that are not considered emblematic by society. This suggestion is in line with findings by Colléony et al (2017) who suggest that zoos should communicate more on the threat levels of species in order to improve social support for their conservation.

Understanding the sociodemographic characteristics that determine social actors’ perceptions towards species can provide useful information to design awareness-raising programs for their conservation. We found that social actors’ environmental behavior, environmental knowledge, and sociodemographic characteristics explain their different perceptions of the different groups of species as emblematic or threatened. For instance, hunters, who hold greater levels of knowledge and frequently visit the area, tended to identify game species and steppe birds as threatened and only games species as emblematic. This result can be explained because hunting steppe birds (from sandgrouses to the great bustard) were banned in the 1980s in Spain (Alonso and Alonso 1996), leading to a general awareness among hunters about the vulnerability of these birds.

In addition, we found that species characteristics could explain differences in perceptions. For example, we found that 34% and 61% of tourists perceive scavengers and predators as emblematic, respectively. These relatively high results can be explained by the greater average body size of species within these functional groups and their availability to be seen compared to the other functional groups of species. By contrast, we found that certain steppe birds, which have smaller body sizes and are more active at dawn and dusk than avian scavengers (more elusive to respondents), were perceived less as emblematic by respondents. These results together support previous research that determines species body size are a key factor for defining emblematic species and influencing social support towards conservation (Clucas et al. 2008; Martín-López et al. 2009; Brooke et al. 2014).

Former research also found that the perception of a species as emblematic can often determine the focus of scientific activities (Wilson et al. 2007) and environmental education programs (Martín-López et al. 2009; Kim et al. 2014; Jarić et al. 2019; Lozano et al. 2019). This finding is supported by our research since Egyptian and griffon vultures, two of the most mentioned species (mentioned by 25% and 37% of respondents, respectively), have been the main focus of long-term monitoring and GPS-tagged research projects in Bardenas Reales Natural Park (Cortés-Avizanda et al. 2015, 2018; Sanz-Aguilar et al. 2017; Arrondo et al. 2020; Donázar et al. 2020). Furthermore, the wild rabbit which was mentioned the most (by 47% of respondents) is one of the species that has attracted a large amount of scientific interest because of its role as a keystone species in the Mediterranean biome (Delibes-Mateos et al. 2007; Cortés-Avizanda et al. 2015). In this context, we argue that more effort is required to draw scientific and societal focus towards rare and elusive steppe birds, such as the Dupont's larks, which is of great conservation concern (Reino et al. 2010; Morales and Traba 2016).

Overall, this research calls for a reassessment of current research and conservation efforts in order to redirect appropriate attention to those, elusive species that are not considered as emblematic and have, thus, potentially received deficient research and conservation attention. In addition, this research highlights the need to consider different social actors when designing conservation actions. Exploring the relationship between social actor characteristics and their perceptions of different species groups can contribute important insights to aid the design of conservation actions that are socially supported.

## Conclusions

This study provides interesting results regarding social actor perceptions of species that may be useful for designing conservation actions that are socially supported in Mediterranean protected areas. We found that in general, perceptions of species as emblematic or threatened vary between social actor groups and are determined by their sociodemographic characteristics, environmental behavior, and knowledge. These results suggest that conservation practitioners and protected area managers need to be attentive to different social actors’ perceptions of biodiversity for the design and implementation of conservation actions. Our study supports previous calls for the integration of social perceptions in the research and management programs of wildlife and protected areas (Bennett 2016; Bennett et al. 2019; Perino et al. 2019; Pascual-Rico et al. 2020). With this study, we go further and call for the inclusion of social perceptions according to different social actor groups and species groups. The development of conservation and education programs that target species need to consider the different perceptions, interests, and knowledge of multiple social actor groups. In addition, these programs should be designed by considering the different perceptions of species groups. Finally, more efforts are required to raise social awareness and support towards those elusive and endangered species not generally viewed as emblematic and in need of research and conservation attention, such as steppe birds.

## Supplementary Information

Below is the link to the electronic supplementary material.

Electronic supplementary material 1 (PDF 344 kb)
