# Supplementary material for: Social actors’ perceptions of wildlife: Insights for the conservation of species in Mediterranean protected areas
Source: Ambio. 2021 Jul 12;51(4):990–1000. doi: 10.1007/s13280-021-01546-6 (PMC8847512; doi:10.1007/s13280-021-01546-6)
Supplement: Supplementary file 1 — Electronic supplementary material 1 (PDF 344 kb) [file 13280_2021_1546_MOESM1_ESM.pdf]

**Electronic Supplementary Material**

This supplementary material has not been peer reviewed.

# Social actors' perceptions of wildlife: Insights for the conservation of species in Mediterranean protected areas

*Running title: Social actors, perceptions and wildlife species*

**Appendix A:**

Definition of all the social actor groups in the study area:

Animal husbandry workers: people engaged in livestock keeping and moving herds (sheep and goats) within the protected area.

Farmers and other locals: farmers whose crops (cereal) are within the protected area and inhabitants from the towns on the border of the study area (within the protected area there are no permanent human settlements). We unite these two categories because, unlike the others, they all live in the towns bordering the park and have special consideration (special treatment) under internal laws.

Hunters: people with a license to hunt in the area during the hunting period.

Tourists: outside visitors not subscribed to any of the above-mentioned categories.

Table S1: Population size (*N*), sample size (*n*), and sampling error for each social actor group.

| **Social actor** | ***N*** | ***n*** | ***Sampling error*** |
|---|---|---|---|
| Animal husbandry workers | 73 | 36 | 6.0% |
| Local inhabitants and Farmers | 4876 | 122 | 4.5% |
| Hunters | 651 | 66 | 5.8% |
| Tourists | 56094 | 130 | 4.4% |
| **Total** | **61394** | **354** | **2.6%** |

**Figure S1:** The effect of the interaction between the explanatory variables social actors and species on the number of species known in Bardenas Reales Natural Park (see model in Table 2).

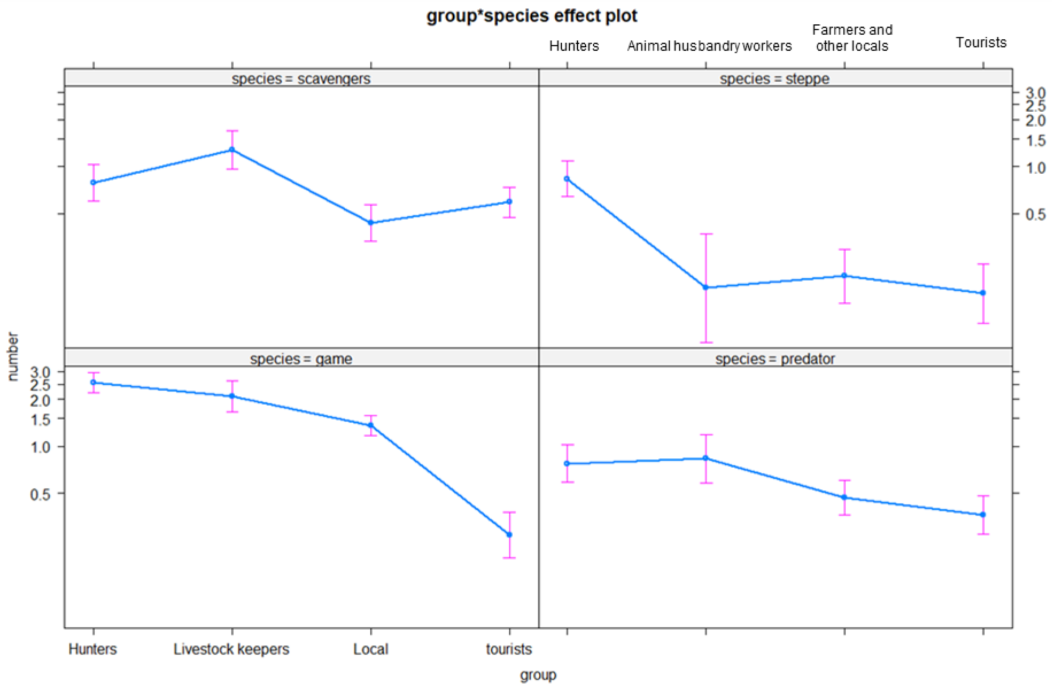

**Appendix B:**

**Questionnaire structure and content**

Nº of questionnaire: ___________

Date:_____________ Hour:________ Site:_____________________________

**I. <u>Data of Respondent</u>**

1.Gender: Male ☐ Female ☐

2. Age: ___________

3. Place of origin: _______________________________________________ (municipality)

4. Time living in the area (for local people): ________________ (years)

**II. <u>Environmental behavior: respondent's relationship with the study area</u>**

5. Is this the first time you have come to Bardenas? Yes ☐ No ☐

(If the answer is No)

How often do you come to Bardenas? _________________________________ (annotate)

When was the last time you came to Bardenas? _________________________ (annotate)

6. What is the main reason for the current visit?

Undecided ☐ Work ☐ Livestock-farmer ☐ Farmer ☐ Hunter ☐

Nature tourism ☐ Rural tourism ☐ Cultural tourism ☐

Others : _________________________________ (annotate)

**III. <u>Respondents' environmental knowledge</u>**

7. How many protected areas have you visited in the last year?_____________ (annotate)

8. Did you know that Bardenas is a protected area since 1999? Yes ☐ No ☐

9. Do you know why there have been measures taken to conserve this area? Yes ☐ No ☐

(if the answer is Yes)

Can you name and explain them?

_____________________________________________________________________________________

_____________________________________________________________________________________

_____________________________________________________________________________________

## IV. Knowledge of species living in the area

10. Do you know the fauna that inhabits this area?

Yes ☐ No ☐

(if the answer is Yes) Can you name the species inhabiting here?

______________________________________________________________________

______________________________________________________________________

## V. Perception of the respondent of the importance and vulnerability of the fauna

11. Do you know if any of the aforementioned species is threatened? Yes ☐ No ☐

(if the answer is Yes) Which ones? ______________________________________________

12. In Bardenas there are several species of game, steppe, predator and avian scavenger species: which group of species do you think are emblematic?

Undecide ☐ None ☐ Game ☐

Steppe ☐ Predators ☐ Avian Scavengers ☐

Others: ________________________(annotate)

13. Which group of species do you think is the most abundant?

Undecide ☐ None ☐ Game ☐

Steppe ☐ Predators ☐ Avian Scavengers ☐

Others: ________________________(annotate)

14. Which group do you think is the most endangered?

Undecide ☐ None ☐ Game ☐

Steppe ☐ Predators ☐ Avian Scavengers ☐

Others: ________________________(annotate)

## Final: Evaluation of the respondent (to fill by the researchers)

Attitude of the respondent Positive ☐ Neutral ☐ Negative ☐

Level of understanding of the respondent High ☐ Medium ☐ Low ☐
